# Comparing empathy in medical students of two Portuguese medicine schools

**DOI:** 10.1186/s12909-020-02034-3

**Published:** 2020-05-13

**Authors:** Luiz Miguel Santiago, Inês Rosendo, Mona-Lisa Coutinho, Katia Sophie Maurício, Isabel Neto, José Augusto Simões

**Affiliations:** 1 Faculty of Medicine of the University of Coimbra, Coimbra, Portugal; 2 General Practice/Family Medicine Clinic of the University of Coimbra, Coimbra, Portugal; 3 CEISUC, Coimbra, Portugal; 4 USF Coimbra Centro, ARS Centro, Coimbra, Portugal; 5 CINTESIS, Porto, Portugal; 6 Faculty of Health Sciences of the University of Beira Interior, Covilhã, Portugal; 7 USF Caminhos do Cértoma, ARS Centro, Coimbra, Portugal

**Keywords:** Medical empathy, Medical students, Doctor-patient relationship, JSPE, Medical education, Student’s empathy

## Abstract

**Background:**

Empathy is paramount in the doctor-patient relationship being a comprehensive and multidimensional concept. Self-ratings of empathy change across the years of medical education only when the Jefferson Scale of Physicians Empathy is used, with a worrying decrease being found throughout the years in Medicine Schools. As there are only few studies on the factors influencing medical student’s empathy, particularly of the curricular model, this study aimed to compare the levels of empathy of medicine students of two Schools with different curricular models in central Portugal, the Faculty of Medicine - University of Coimbra (FMUC) and the Faculty of Health Sciences - University of Beira Interior (FCS-UBI).

**Methods:**

Cross-sectional observational study with the Jefferson Scale of Physician Empathy – students’ Portuguese version (JSPE – spv) to 1st, 3rd and 6th year students of the 2017/2018 academic year with descriptive and inferential statistical analysis (*p* < 0.05).

**Results:**

Size representative sample of 795 students. Higher total empathy score (TES) (*p* = 0.008) and “Perspective taking” (*p* = 0.001) in FCS-UBI were found. JSPE-TES was higher in FCS-UBI, 3rd year (*p* = 0.038). Higher FCS-UBI “Perspective taking” in the 1st year (*p* = 0.030) and 6th year (*p* = 0.044), for “Compassionate care” in the 3rd (*p* = 0.019) and for “Standing in the patient’s shoes” in the 1st year (*p* = 0.018) and in FMUC for “Compassionate care” in the 1st year (*p* = 0.037) and the “Standing in the patient’s shoes” in year 3 (*p* = 0.002) were found. Higher levels of empathy were found in FCS-UBI female students, for JSPE-TES (*p* = 0.045) and “Perspective taking” (*p* = 0.001).

**Conclusion:**

Higher empathy levels in FCS-UBI were found, with different results in the third year suggesting influence of the medical course teaching characteristics between the two Medicine schools, student’s empathy levels being higher when earlier and more intense contact with patients accompanied by skilled tutors was developed.

## Background

Empathy is paramount in the doctor-patient relationship, contributing to patient satisfaction with their doctor, ease in providing relevant information, better adherence to therapy and clinical results, lower litigation rates for medical mal-practice and levels of stress and professional burnout [1–4].

Empathy is a comprehensive and multidimensional concept. Hojat et al. have described it as a predominantly cognitive (rather than emotional) attribute that involves the ability to understand (rather than feel) the patient’s experiences, concerns, and perspectives associated with the ability to communicate that same understanding [5].

Empathy can be taught and trained through educational processes and reflection [6–9].

Thus, it is important to promote effective educational interventions that can improve and maintain the levels of empathy in students, physicians to be as well in practicing doctors, contributing to the strengthening of the doctor-patient relationship and to a better healthcare system [6, 8, 9].

Despite the consensus on the importance of empathy in medical education and practice [6], a worrying decrease in the empathy of medical students throughout their years in Medicine Schools is reported [6, 10–14]. A recent meta-analysis indicates significant evidence of self-ratings of empathy changing across the years of medical education only when the JSPE is used [9].

Scarce Portuguese literature focused on the evolution of empathy in portuguese medical students. A multi-institutional study reveals that empathy and personality of medical students are closely related [7]. Cross-cultural adaptation and validation of the Jefferson Scale of Physician Empathy – students’ Portuguese version (JSPE – spv) has already been made [1, 5, 15].

There are few studies on the factors that can influence the empathy of medical students, particularly the influence of the curricular model practiced in the different Medicine Schools [2].

This study aimed to compare the levels of empathy of medicine students of two Schools with different curricular models, allowing investigation on the differences and influences of the teaching system in student’s empathy.

We compared the Faculty of Medicine of the University of Coimbra (FMUC) and the Faculty of Health Sciences of the University of Beira Interior (FCS-UBI).

To put it into context, the two Medicine Schools have the same admission process for incoming students, most of them admitted through a competitive national exam [16, 17], females entering more in both schools at an approximate 3:1 ratio, higher marks being observed for entrande at FMUC than at FCS-UBI. FMUC receiving 350 students a year and FCS-UBI 150. In both Schools, the course is 6 years long [16, 17] but the student-tutor ratio is higher at FMUC 18.5 for FMUC and 3.1 for FCS-UBI [17, 18]. FMUC’s teaching is semesterial, applies a non-integrated teaching system, based on the compartmentalization of contents into subjects, emphasizes the “storage” of information [16, 18, 19] and evaluations are mainly by theoretical exams. FCS-UBI has a quarterly period organization and applies an integrated teaching system promoting self-directed learning using, whenever possible, problem-based learning [17, 20]. According to European “Bologna Declaration” [https://ec.europa.eu/education/policies/higher-education/bologna-process-and-european-higher-education-area_pt accessed on the 19th December 2019] medicine students are to perform a Thesis at the end of their studies, so acquiring a Master on Medicine degree (MIM).

Details on curricula information can be obtained ate the Medicine School’s website [16, 17, 19, 20]. In brief, analysing the curriculum of each faculty, FCS-UBI includes more contents in the area of human sciences, more role-playing activities and an earlier clinical in-practice contact with patients [8, 21, 22].

This study objectives were to understand the differences iuniversities n levels of empathy between FMUC and FCS-UBI medical students, and to compare them by gender and scholar year.

## Methods

We performed a cross-sectional observational study applying an instrument to measure the levels of empathy of first, third and sixth-year students attending the MIM in FMUC and FCS-UBI after approval by the Ethics Committee for Health of the Central Regional Health Administration of Portugal was granted.

The JSPE-spv, a self-administered questionnaire allowing evaluation of the student’s self-perception of empathy in patient care, was used [5]. It is considered a reliable instrument with evidence that supports its validity [1, 5, 15, 23, 24]. With 20 items, to be answered on a Likert scale from 1 (strongly disagree) to 7 points (strongly agree) it studies three components: “Perspective taking” (10 items; refers to the ability to analyse another person’s problem from the outside), “Compassionate care” (8 items, defined by the activity in favour of the one we see suffering) and “ Standing in the patient’s shoes” (2 items; refers to the act of thinking as if we were in the other person’s place) [5, 15, 23]. The total score varies from 20 to 140, and the higher the score the higher the level of empathy [15, 23].

The sample size was calculated after the total number of students to be enrolled in each faculty in the academic year 2017/2018 per year of attendance. FMUC 471 students in the 1st year, 328 in the 3rd year and 314 in the 6th year and FCS-UBI 186 in the 1st year, 136 in the 3rd year and 141 in the 6th year. The sample size calculation was made using the site “The Survey System - Sample Size Calculator” [25] to represent the universe with a 95% confidence level and a 5% margin of error.

The study instrument was distributed in paper during practical classes of the first, third and sixth curricular year in both universities, in 2018 April and May. Responsible Professors for each randomly chosen class were previously informed and granted consent. The number of students to be enrolled, implied a draw of each year classes to achieve the needed sample size, with extra classes for sample collection security reasons. Student’s participation was individual, voluntary, anonymous and with written informed consent. Information on gender (male / female) and MIM year (first / third / sixth) was obtained.

The SPSS®) Software for Windows version 24.0″ was used for storage, descriptive and inferential statistical analysis of data. Checking the non-normality of the data distribution with the Kolmogorov-Smirnov test, non-parametric tests (Mann-Whitney test) was used for statistical analysis. A *p* value of < 0.05 was considered for significant difference.

## Results

A representative sample size of at least 286 questionnaires at FMUC and 210 at FCS-UBI was estimated. For this study a sample of 795 medical students, 420 from FMUC and 375 from FCS-UBI was studied. Its description according to the university, the MIM year attended and gender is shown in Table 1. The Kolmogorov-Smirnov test showed no numeric data with normal distribution (*p* < 0.001). Using the chi-square test and the Mann-Whitney U test, no significant differences were found between universities for gender (*p* = 0.096) and year of MIM (*p* = 0.408).

**Table 1:** Sample according to University, year of frequency of Master In Medicine (MIM) and gender

	FMUC n (%)	FCS-UBI n (%)	Total n (%)
**Year of MIM (*p*** **= 0,408)**^**a**^			
1°	152 (36.2)	153 (40.8)	305 (38.4)
3°	164 (39.0)	137 (36.5)	301 (37.9)
6°	104 (24.8)	85 (27.7)	189 (23.8)
Total	420 (100)	375 (100)	795 (100)
**Gender (*p*** **= 0,096)**			
Female	278 (69.8)	282 (75.2)	560 (72.4)
Male	120 (30.2)	93 (24.8)	213 (27.6)
Total	398 (100)	375 (100)	773 (100)

*FMUC* Faculty of Medicine of the University of Coimbra, *FCS-UBI* Faculty of Health Sciences of the University of Beira Interior. (*) Mann-Whitney U

Table 2 shows the differences in the mean value of the answers scores between universities for total JSPE and each one of its components (“Perspective taking,” “Compassionate care”, “Standing in the patient’s shoes”).

**Table 2:** Differences in the mean value of the answers per university for the total JSPE and for each one of its components “Perspective taking”, “Compassionate care” and “Standing in the patient’s shoes” by University

	FMUC	FCS-UBI	p (*)
**JSPE total (Max = 140)**			
N	420	375	0.008
Mean ± SD	89.2 ± 7.6	90.6 ± 7.6	
95% ic	88,2 to 89,14	89,75 to 91,34	
**Perpective taking (Max = 70)**			
N	420	375	0.001
Mean ± SD	59.4 ± 5.8	60.7 ± 6.7	
95% ic	58,44 to 59,21	60,04 to 61,39	
**Compassionate care (Max = 56)**			
N	420	375	0.800
Mean ± SD	15.1 ± 4.1	15.2 ± 4.3	
95% ic	14,71 to 15,21	14,75 to 15,61	
**Standing in the patient’s shoes (Max = 14)**			
N	420	375	0.399
Mean ± SD	7.4 ± 2.3	7.3 ± 2.5	
95% ic	7,32 to 7,59	7,01 to 7,52	

*FMUC* Faculty of Medicine of the University of Coimbra, *FCS-UBI* Faculty of Health Sciences of the University of Beira Interior. (*) Mann-Whitney U

With the Mann-Whitney U test significant differences were found between the two Schools for the overall JSPE score (*p* = 0.008) and the “Perspective taking” component score (*p* = 0.001) with a higher score in FCS-UBI.

Table 3 reveals the mean value of the responses per Medicine School and per year of MIM for the total JSPE and for each of its components per year between Scholls (“Perspective taking”, “Compassionate care”, “ Standing in the patient’s shoes”) and for gender, again with Mann-Whitney U test. There was significant difference in the 3rd year (*p* = 0.038), higher score in the FCS-UBI.

**Table 3:** Differences in the mean value of the responses per university and per year of MIM and gender for the total JSPE and for each of its components “Perspective taking”, “Compassionate care” and “Standing in the patient’s shoes”

Year of attendance	FMUC			FCS-UBI			p (between universities) (*)		
	1°	3°	6°	1°	3°	6°	1°	3°	6°
**JSPE total (Max = 140)**									
N	152	164	104	153	137	85	0.215	0.038	0.308
Mean ± SD	90.3 ± 9.1	90.4 ± 5.7	85.7 ± 6.9	91.8 ± 7.4	91.5 ± 7.6	86.8 ± 7.1			
**Perpespective taking (Max = 70)**									
N	152	164	104	153	137	85	0.030	0.108	0.044
Mean ± SD	59.0 ± 6.1	60.4 ± 5.6	58.5 ± 5.8	60.8 ± 7.3	61.0 ± 6.5	60.1 ± 5.8			
**Compassionate care (Max = 56)**									
N	152	164	104	153	137	85	0.037	0.019	0.741
Mean ± SD	16.8 ± 4.5	13.9 ± 3.6	14.5 ± 3.1	15.9 ± 5.0	14.9 ± 3.7	14.5 ± 3.6			
**Standing in the patient’s shoes (Max = 14)**									
N	152	164	104	153	137	85	0.018	0.002	0.611
Mean ± SD	6.6 ± 2.3	8.2 ± 2.2	7.2 ± 2.2	7.3 ± 2.4	7.4 ± 2.6	7.0 ± 2.5			
**Gender**	**Female**	**Male**	**Female**		**Male**	**Female**		**Male**	
**JSPE total (Maximum = 140)**									
N	278	120	282		93	0.045		0.071	
Mean ± SD	89.0 ± 8.1	89.8 ± 6.8	90.4 ± 7.7		91.0 ± 7.4				
**Perspective taking (Max = 70)**									
N	278	120	282		93	0.001		0.777	
Mean ± SD	59.7 ± 5.7	58.9 ± 6.1	61.3 ± 6.4		58.8 ± 7.0				
**Compassionate care (Max = 56)**									
N	278	120	282		93	0.874		0.174	
Mean ± SD	14.8 ± 3.9	15.9 ± 4.1	14.7 ± 4.1		16.6 ± 4.4				
**Standing in the patient’s shoes (Max = 14)**									
N	278	120	282		93	0.321		0.704	
Mean ± SD	7.3 ± 2.4	7.6 ± 2.3	7.1 ± 2.5		7.7 ± 2.4				

*FMUC* Faculty of Medicine of the University of Coimbra, *FCS-UBI* Faculty of Health Sciences of the University of Beira Interior. (*) Mann-Whitney U

For “Perspective taking” there were significant differences in the 1st (*p* = 0.030) and in the 6th year (*p* = 0.044), both scores being higher in the FCS-UBI.

For the “Compassionate care” component, there was a difference in the 1st year (*p* = 0.037), and in the 3rd year with higher scores in the FCS-UBI (*p* = 0.019).

Regarding the “Standing in the patient’s shoes” component, significant differences were found in the 1st year (*p* = 0.018), FCS-UBI scoring better, and in the 3rd year (*p* = 0.002) higher at the FMUC.

By gender between universities for total JSPE and for each of its components there was a statistically significant difference, better for female students, for the total JSPE score (*p* = 0.045) and the “Perspective taking” score (*p* = 0.001), again with a higher score in FCS-UBI.

## Discussion

Significantly higher levels of empathy were found in FCS-UBI students for the total score of JSPE, for the “Perspective taking” component and for 3rd year students still with higher scores in FCS-UBI.

These results are in line with the only study we found comparing the levels of empathy in two universities with different curricular models in a Pakistan study [26]. Another multi-institutional study revealed the influences of teaching scheme on the levels of medical student’s empathy though no specification of curricular model was reported [27]. Given the different socio-economic environments and medical and educational contexts between Portugal and Pakistan we can only recognise that Portuguese medicine students appear to be less empathic, the rasons for such being speculative.

Lower levels of empathy in FMUC students can possibly be explained by:

Teaching characteristics of each school.

FCS-UBI curriculum providing in-practice classes with the student accompanied by skilled tutor together with practical sessions following a theoretical tutorial.

Analysing the curricula of both University Medical schools [16, 17]. FCS-UBI had a larger number of curricular units related to humanistic sciences, with emphasis on the development of interaction and communication skills. Role-playing activities are conducted regularly with discussion and specific assessment of students’ communicative and empathic abilities is made [8, 21, 22]. FMUC lacks subjects related to the humanistic sciences and has a more superficial and limited approach to clinical practice started in the 4th year of MIM. The curricular unit of General Practice/Family Medicine, is one of the two curricular units to include the discussion of topics such as empathy and communication and the performance of role-playing activities, is only taught in the 5th year. So by the 3rd year, FCS-UBI students already have had contact with clinical practice, whereas FMUC students have not [16, 17]. Tutor’s characteristics influence the levels of student’s empathy [14, 21]. Thus, the differences between the two universities can also be so explained, and that deserves future study, supporting interventions in their tutors, upgrading them.

Other secondary factors can also help explain different levels of epathy:

Student-tutor ratio FMUC with a ratio of 18.5 (Portuguese average 7.53), contrasting with a 3.1 ratio in FCS-UBI. Higher student-tutor ratio at FMUC contributed to lower satisfaction with teaching, less clinical contact and less opportunities to develop empathic relationships with patients [18]. Author data affirm the need of adequate role-models for empathy. In fact FMUC students have a 13 ECTS content in General Practice ate the 1st year, 6 h, 5th year one semester and 6th year 2 months with a dedicated General Practice tutor, comparing to 30 ECST in FCS-UBI, 1st, 2nd, 4th, 5th and 6th years and do most of their in-practice cases in a large over-crowded Central Hospital [16, 17, 19, 20].

Sudent’s satisfaction: A student’s opinions pool, found out that FMUC was the national medicine school with the worst level of overall satisfaction and clinical teaching and study conditions, whereas FCS-UBI had the highest levels [28].

So far no studies in specific student’s characteristics were performed to differentiate between FMUC and FCS-UBI except for entry marks, higher in Coimbra. Significant differences were only found in feminine gender and only in the total JSPE score and in the “Perspective taking” component, being higher in FSC-UBI, female students of FCS-UBI had higher levels of empathy than male ones.

Empathy is positively influenced by quality of life and negatively by fatigue, stress, and burnout. Studies carried out in both universities that partook in this study showed that, FCS-UBI students have a better quality of life and are less vulnerable to stress and fatigue [28, 29].

The existence of differences in empathy between the two universities supports the need to consider the impact of the curriculum model and other MIM characteristics on the development of the empathic capacities of medical students. So several changes can be considered in order to increase the levels of empathy in medical students, in the long term, in order to improve and maintain the levels of empathy in medical students:

Educational interventions focused on empathic capacities by role-playing, video-watching and real consultations with patients with analysis and discussion of medical communication is deemed necessary [22]. Also lectures and practice of the importance of empathy and communication in the doctor-patient relationship and early integration of more contents of the human sciences area into the study plan, with a more reduced student-tutor ratio, preferably a one to one even if for shorter period of time [8, 9]. Even though “empathy is related to personality”, a matter this study did not focus on [30, 31].

Research is to be continued in this area.

In spite of the attempts to minimize bias, there may have been distraction in reading the questionnaires. Attempts were made to close the selection bias through the random selection of the classes included in the study. The JSPE student’s version evaluates self-perception of empathy, which may be different from the actual empathic behaviour.

As practical points from our study we must emphasize that (1) empathy is a paramount element of the doctor-patient relationship that can be trained through an educational process, (2) student’s empathy levels were higher when earlier and more intense contact with patients accompanied by skilled tutors was developed and (3) reassessment of curricular particularities must bear in mind practical activities in real world context, recognizing that each student has its own intrinsic psychologic characteristics.

## Conclusion

The levels of empathy of FCS-UBI medical students were higher than those found in FMUC students.

Comparing the results per year of MIM, the difference was mainly in the 3rd year with better results in FCS-UBI.

By gender females score statistically different between the two Schools, better in FCS-UBI.

These results support the need to reassess the learning curriculum and the teaching context, with the objective of promoting effective educational interventions in the development of student empathy.

## Data Availability

All data will become available if requested.

## Abbreviations

MIM: Integrated Master’s degree in Medicine
FMUC: Faculty of Medicine of the University of Coimbra
FCS-UBI: Faculty of Health Sciences of the University of Beira Interior
JSPE – spv: Jefferson Scale of Physician Empathy – students’ Portuguese version
TES: total empathy score

## References

[1] Loureiro J, Gonçalves-Pereira M, Trancas B, Caldas-De-Almeida JM, Castro-Caldas A (2011). Empathy in the doctor-patient relationship as viewed by first-year medical students: data on validity and sensibility to change of the Jefferson measure in Portugal. Acta Medica Port.

[2] Hegazi I, Wilson I (2013). Maintaining empathy in medical school: it is possible. Med Tech.

[3] Kim S, Kaplowitz S, Johnston M (2004). The effects of physician empathy on patient satisfaction and compliance. Eval Health Prof.

[4] von Harscher H, Desmarais N, Dollinger R, Grossman S, Aldana S (2017). The impact of empathy on burnout in medical students: new findings. Psychol Health Med.

[5] Hojat M, Vergare MJ, Maxwell K (2009). The devil is in the third year: a longitudinal study of erosion of empathy in medical school. Acad Med.

[6] Costa P, Alves R, Neto I, Marvão P, Portela M, Costa MJ (2014). Associations between medical student empathy and personality: a multi-institutional study. PLoS One.

[7] Hojat M, Axelrod D, Spandorfer J, Mangione S (2013). Enhacing and sustaining empathy in medical students. Med Teach.

[8] Magalhães E, Dechamplain A, Salgueira A, Costa MJ (2010). Medical empathy: Adaptation and validation of a scale for medical students. Book of Abstracts of the 7th National Symposium of Research in Psychology, Braga, Portugal.

[9] Spatoula V, Panagopoulou E, Montgomery A (2019). Does empathy change during undergraduate medical education? – a meta-analysis. Med Teach.

[10] Machado A. Medical empathy in the eyes of medical students [dissertation]. Coimbra (PT): Coimbra University; 2016. (Master’s Tesis in Portuguese).

[11] Park K, Roh H, Suh D, Hojat M (2014). Empathy in Korean medical students: findings from a nationwide survey. Med Teach.

[12] Chen D, Lew R, Hershman W, Orlander J (2007). A cross-sectional measurement of medical student empathy. J Gen Intern Med.

[13] Youssef F, Nunes P, As B, Williams S (2014). An exploration of changes in cognitive and emotional empathy among medical students in the Caribeean. Int J Med Educ.

[14] Neumann M, Edelhäuser F, Tauschel D (2011). Empathy decline and its reasons: a systematic review of studies with medical students and residents. Acad Med.

[15] Aguiar P, Salgueira A, Frada T, Costa MJ (2009). Medical empathy: translation, validation and application of a measurement instrument. Proceedings of the 10th Galician-Portuguese International Congress of Psychopedagogy, Braga, Portugal.

[16] University of Coimbra. Integrated Masters in Medicine. Available in https://apps.uc.pt/courses/en/course/5841. Accessed 20 Jan 2019.

[17] University of Beira Interior. Integrated Masters in Medicine. Available in http://www.ubi.pt/en/course/52. Accessed 20 Jan 2019.

[18] Grilo D, Moreira M, Coimbra A (2016). Study on Portuguese medical Schools' learning conditions: a National Analysis on student satisfaction, student-tutor ratios and number of admissions. Acta Medica Port.

[19] University of Coimbra. Integrated Masters in Medicine. Study Programme. Available in https://apps.uc.pt/courses/en/programme/5841/2018-2019?id_branch%20=16221. Accessed 20 Jan 2019.

[20] University of Beira Interior. Integrated Masters in Medicine. Study Plan. Available in https://www.ubi.pt/en/studyplan/52/1595/2019/. Accessed 20 Jan 2019.

[21] Ahrweiler F, Neumann M, Goldblatt H, Hahn E, Scheffer C (2014). Determinants of physician empathy during medical education: hypothetical conclusions from an exploratory qualitative survey of practicing physicians. BMC Med Educ.

[22] Quince T, Thiemann P, Benson J, Hyde S (2016). Undergraduate medical students' empathy: current perspectives. Adv Med Educ Pract.

[23] Hojat M, Mangione S, Nasca TJ, Cohen MJM, Gonnella JS, Erdmann JB (2011). The Jefferson scale of physician empathy: development and preliminary psychometric data. Educ Psychol Meas.

[24] Hojat M, Gonnella J (2015). Eleven years of data on the Jefferson scale of empathy- medical student version (JSE-S): proxy norm data and tentative cutoff scores. Med Princ Pract.

[25] The Survey System. Sample Size Calculator. Available in https://www.surveysystem.com/sscal.htm. Accessed 2 Feb 2018.

[26] Ayub A, Khan RA (2017). Measuring empathy of medical students studying different curricula; a causal comparative study. J Pak Med Assoc.

[27] Quince TA, Kinnersley P, Hales J (2016). Empathy among undergraduate medical students: a multi-Centre cross-sectional comparison of students beginning and approaching the end of their course. BMC Med Educ.

[28] Pereira APM. Quality of life and vulnerability to stress in 5th and 6th year medical students. [dissertation]. Coimbra (PT): Coimbra University; 2017. http://hdl.handle.net/10316/82616. (Master’s Tesis in Portuguese).

[29] Felizardo MGG, Santiago LM (2019). Quality of life and stress vulnerability in students of the 6th year of the FCS-UBI MIM. Rev ADSO.

[30] Guilera T, Batalla I, Forné C, Soler-González J (2019). Empathy and big five personality model in medical students and its relationship to gender and specialty preference: a crosssectional study. BMC Med Educ.

[31] Shapiro J, Morrison E, Boker J (2004). Teaching empathy to first year medical students: evaluation of an elective literature and medicine course. Educ Health.

